# Inflamação e Nefropatia Associada ao Contraste: O Papel Emergente da Razão Glicose/Linfócitos

**DOI:** 10.36660/abc.20250424

**Published:** 2025-08-20

**Authors:** Rafaela Andrade Penalva Freitas

**Affiliations:** 1 Instituto Dante Pazzanese de Cardiologia São Paulo SP Brasil Instituto Dante Pazzanese de Cardiologia, São Paulo, SP – Brasil

**Keywords:** Nefropatias, Inflamação, Infarto do Miocárdio

A relação entre inflamação sistêmica, metabolismo glicêmico e desfechos cardiovasculares tem sido amplamente estudada na literatura, com implicações na prática clínica. O artigo intitulado "Associação entre Relação Glicose/Linfócito e Lesão Renal Aguda Induzida por Contraste em Pacientes com Infarto do Miocárdio Não Diabéticos" oferece uma contribuição ao investigar a razão glicose/linfócitos (GLR) como um preditor independente de nefropatia induzida por contraste (NIC) em pacientes com infarto agudo do miocárdio com supradesnivelamento do segmento ST (IAMCSSST) submetidos à intervenção coronária percutânea primária (ICP).^1^ Este editorial analisa os achados do estudo, contextualiza sua importância e discute implicações clínicas e futuras para a pesquisa.

A NIC é uma complicação que pode ocorrer após procedimentos que utilizam meios de contraste iodados com incidência variando de 5% a 20% em populações com IAMCSSST. Essa condição está associada a piores desfechos, incluindo maior mortalidade e progressão para doença renal crônica.^2 , 3^ Fatores como inflamação sistêmica e estresse metabólico, frequentemente exacerbados no contexto do IAM, desempenham papéis importantes na patogênese da NIC.^4 , 5^ O estudo em questão introduz a GLR como um biomarcador composto que integra informações sobre metabolismo glicêmico e resposta inflamatória, oferecendo uma abordagem inovadora para estratificação de risco.^1^

A incidência global de NIC foi de 7,4%, mas o grupo com GLR elevada apresentou uma incidência significativamente maior (30,9% vs. 1,3% no grupo com GLR baixa, p<0,001). Após ajustes, a GLR elevada permaneceu como um preditor independente de NIC, assim como a creatinina basal. Esses achados sugerem que a GLR pode capturar sinergias entre inflamação e estresse metabólico, fornecendo uma ferramenta prognóstica robusta.^1^

A inflamação desempenha papel central na patogênese da NIC. O meio de contraste iodado pode induzir lesão endotelial, hipóxia tubular e liberação de espécies reativas de oxigênio, amplificando a resposta inflamatória.^6^ No contexto do IAMCSSST, a liberação de citocinas pró-inflamatórias, como interleucina-6 e fator de necrose tumoral alfa, exacerba estresse oxidativo e disfunção renal.^5 , 6^ A linfocitopenia relativa, frequentemente observada em estados inflamatórios agudos, reflete a redistribuição de linfócitos para tecidos inflamados ou apoptose induzida por estresse.^7^ Por outro lado, a hiperglicemia de estresse, promove disfunção endotelial e inflamação através da ativação de vias como a da proteína quinase C e da formação de produtos finais de glicação avançada.^7^

A GLR oferece uma métrica integrativa que reflete a interação entre inflamação sistêmica e desregulação metabólica. A força do estudo reside na sua capacidade de demonstrar que a GLR elevada não apenas está associada a um risco significativamente maior de NIC, mas também mantém sua significância após ajuste para fatores de confusão, como creatinina basal. Isso sugere que a GLR pode capturar aspectos da fisiopatologia da NIC que vão além dos preditores tradicionais, como função renal pré-existente.^1^

A identificação de biomarcadores simples e acessíveis é particularmente relevante em cenários de emergência, onde o tempo para decisão clínica é limitado. A GLR pode ser facilmente calculada a partir de exames laboratoriais de rotina (glicemia e contagem de linfócitos), tornando-a ferramenta prática para estratificação de risco na admissão. Pacientes com GLR elevada poderiam se beneficiar de estratégias preventivas, como hidratação e minimização do volume de contraste.^3 , 8 - 10^ A GLR também pode ajudar a identificar subgrupos de pacientes que requerem monitoramento mais rigoroso da função renal.

Embora promissor, o estudo apresenta limitações que merecem consideração. Sua natureza retrospectiva e unicêntrica pode limitar a generalização dos resultados. A exclusão de pacientes com diabetes mellitus, embora justificada para evitar confusão, restringe a aplicabilidade dos achados a uma população mais ampla. Estudos prospectivos e multicêntricos são necessários para validar a GLR como um biomarcador universal para NIC em diferentes contextos clínicos. A falta de dados sobre outros biomarcadores inflamatórios, como proteína C reativa ou interleucinas, que poderiam fornecer dados adicionais sobre os mecanismos subjacentes à associação entre GLR e NIC. Além disso, volume de contraste utilizado ou tipo de contraste (iso-osmolar vs. baixa-osmolaridade), não foi explorada em profundidade, o que poderia refinar ainda mais a utilidade clínica da GLR.^3 , 9^

Pesquisas futuras também devem investigar se a GLR pode prever outros desfechos adversos em pacientes com IAMCSSST, como mortalidade a longo prazo ou re-hospitalização. A integração da GLR com escores de risco existentes, como o *Mehran Risk Score* para NIC, pode melhorar a acurácia preditiva.^4 , 10 , 11^ Finalmente, a validação da GLR em populações com diferentes comorbidades, é importante para estabelecer sua robustez como biomarcador global.

Este estudo representa um avanço significativo na compreensão da interação entre inflamação, estresse metabólico e lesão renal no contexto do IAMCSSST. A GLR emerge como um biomarcador promissor, acessível e clinicamente relevante, com potencial para melhorar a estratificação de risco e orientar intervenções preventivas. A validação em estudos prospectivos e integração com outras ferramentas diagnósticas são passos importantes para consolidar seu papel na prática clínica. Este trabalho destaca a importância de abordagens integrativas na cardiologia, unindo marcadores metabólicos e inflamatórios.
