# Supplementary material for: Inflamação e Nefropatia Associada ao Contraste: O Papel Emergente da Razão Glicose/Linfócitos
Source: Arq Bras Cardiol. 2025 Aug 20;122(8):e20250424. [Article in Portuguese] doi: 10.36660/abc.20250424 (PMC12671837; doi:10.36660/abc.20250424)
Supplement: Translation

# Translation: Inflammation and Contrast-Induced Nephropathy: The Emerging Role of the Glucose-to-Lymphocyte Ratio

**DOI:** 10.36660/abc.20250424i

**Authors:** Rafaela Andrade Penalva Freitas

**Affiliations:** 1 São Paulo SP Brazil Instituto Dante Pazzanese de Cardiologia, São Paulo, SP – Brazil

**Keywords:** Kidney Diseases, Inflammation, Myocardial Infarction

The interplay between systemic inflammation, glucose metabolism, and cardiovascular outcomes has been extensively explored in the literature, with significant implications for clinical practice. The article titled "Association between Glucose/lymphocytes Ratio and ContrastInduced Acute Kidney Injury in Patients with Myocardial Infarction without Diabetes Mellitus" provides a valuable contribution by investigating the glucose-to-lymphocyte ratio (GLR) as an independent predictor of contrast-induced acute kidney injury (CI-AKI) in patients with ST-elevation myocardial infarction (STEMI) undergoing primary percutaneous coronary intervention (PCI).^1^This editorial examines the study's findings, contextualizes their significance, and discusses clinical implications and future research.

CI-AKI is a significant iatrogenic complication following procedures involving iodinated contrast media, with reported incidence rates ranging from 5% to 20% in STEMI populations. This condition is associated with worse outcomes, including higher mortality and progression to chronic kidney disease.^2 , 3^Factors such as systemic inflammation and metabolic stress, often exacerbated in the context of acute myocardial infarction (AMI), play critical roles in the pathogenesis of CI-AKI.^4 , 5^The study introduces the GLR as a composite biomarker that integrates information on glucose metabolism and inflammatory response, offering an innovative approach to risk stratification.^1^

The overall CI-AKI incidence was 7.4%, but the high-GLR group (≥4.16) exhibited a significantly higher incidence (30.9% vs. 1.3% in the low-GLR group, p<0.001). After adjusting for confounders, high GLR remained an independent predictor of CI-AKI, as did baseline creatinine. These findings suggest that GLR may capture synergies between inflammation and metabolic stress, providing a robust prognostic tool.^1^

Inflammation plays a central role in the pathogenesis of CI-AKI. Iodinated contrast media can induce endothelial injury, tubular hypoxia, and the release of reactive oxygen species, amplifying the inflammatory response.^6^In the context of STEMI, the release of pro-inflammatory cytokines, such as interleukin-6 and tumor necrosis factor-alpha, exacerbates oxidative stress and renal dysfunction.^5 , 6^Relative lymphocytopenia, often observed in acute inflammatory states, reflects lymphocyte redistribution to inflamed tissues or stress-induced apoptosis.^7^Conversely, stress hyperglycemia, common in AMI patients, promotes endothelial dysfunction and inflammation through pathways such as protein kinase C activation and the formation of advanced glycation end-products.^7^

By combining these two parameters, GLR offers an integrative metric that reflects the interplay between systemic inflammation and metabolic dysregulation. The study's strength lies in its ability to demonstrate that elevated GLR is not only associated with a significantly higher risk of CI-AKI but also retains its significance after adjustment for confounders, such as baseline creatinine. This suggests that GLR may capture aspects of CI-AKI pathophysiology beyond traditional predictors, such as pre-existing renal function.^1^

The identification of simple and accessible biomarkers, such as GLR, is particularly relevant in emergency settings like STEMI, where clinical decision-making time is limited. GLR can be easily calculated from routine laboratory tests (blood glucose and lymphocyte count), making it a practical tool for risk stratification upon admission. Patients with elevated GLR could benefit from intensive preventive strategies, such as aggressive hydration and minimization of contrast volume.^3 , 8 - 10^Additionally, GLR may help identify subgroups of patients requiring closer monitoring of renal function post-PCI.

While promising, the study has limitations that warrant consideration. Its retrospective, single-center design may limit the generalizability of the findings. Furthermore, the exclusion of patients with diabetes mellitus, though justified to avoid confounding by chronic hyperglycemia, restricts the applicability of the results to a broader population. Prospective, multicenter studies are needed to validate GLR as a universal biomarker for CI-AKI across diverse clinical contexts, including diabetic patients. Another limitation is the lack of data on other inflammatory biomarkers, such as C-reactive protein or interleukins, which could provide additional insights into the mechanisms underlying the association between GLR and CI-AKI. Moreover, the interaction between GLR and other CI-AKI risk factors, such as contrast volume or type (iso-osmolar vs. low-osmolar), was not thoroughly explored, which could further refine GLR's clinical utility.^3 , 9^

Future research should also investigate whether GLR can predict other adverse outcomes in STEMI patients, such as long-term mortality or rehospitalization. Integrating GLR with existing risk scores, such as the Mehran Risk Score for CI-AKI, may enhance predictive accuracy and personalize preventive strategies.^4 , 10 , 11^Finally, validating GLR in more diverse populations, including those with varying comorbidities, is essential to establish its robustness as a global biomarker.

The study on the association between GLR and CI-AKI represents a significant advance in understanding the interplay between inflammation, metabolic stress, and renal injury in the context of STEMI. GLR emerges as a promising, accessible, and clinically relevant biomarker with the potential to improve risk stratification and guide preventive interventions. However, its validation in prospective studies and integration with other diagnostic tools are critical steps to solidify its role in clinical practice. This work underscores the importance of integrative approaches in cardiology, combining metabolic and inflammatory markers to address complex challenges like CI-AKI.
